# Text-messaging versus telephone reminders to reduce missed appointments in an academic primary care clinic: a randomized controlled trial

**DOI:** 10.1186/1472-6963-13-125

**Published:** 2013-04-04

**Authors:** Noelle Junod Perron, Melissa Dominicé Dao, Nadia Camparini Righini, Jean-Paul Humair, Barbara Broers, Françoise Narring, Dagmar M Haller, Jean-Michel Gaspoz

**Affiliations:** 1 Division of primary care medicine, Department of community medicine, primary care and emergency medicine, Geneva University Hospitals, 4 rue Gabrielle Perret-Gentil, Geneva 14, 1211, Switzerland; 2 Fondation Phenix, Rue du Grand-Pré 72, Geneva, 1202, Switzerland; 3 Adolescent&Young Adult Program, Department of pediatrics, Geneva University Hospitals, rue Gabrielle Perret-Gentil, Geneva 14, 1211, Switzerland

**Keywords:** Reminders, Telephone, Text-message, Missed appointments, Primary care, Randomized controlled trial

## Abstract

**Background:**

Telephone or text-message reminders have been shown to significantly reduce the rate of missed appointments in different medical settings. Since text-messaging is less resource-demanding, we tested the hypothesis that text-message reminders would be as effective as telephone reminders in an academic primary care clinic.

**Methods:**

A randomized controlled non-inferiority trial was conducted in the academic primary care division of the Geneva University Hospitals between November 2010 and April 2011. Patients registered for an appointment at the clinic, and for whom a cell phone number was available, were randomly selected to receive a text-message or a telephone call reminder 24 hours before the planned appointment. Patients were included each time they had an appointment. The main outcome was the rate of unexplained missed appointments. Appointments were not missed if they were cancelled or re-scheduled before or independently from the intervention. We defined non-inferiority as a difference below 2% in the rate of missed appointments and powered the study accordingly. A satisfaction survey was conducted among a random sample of 900 patients (response rate 41%).

**Results:**

6450 patients were included, 3285 in the text-message group and 3165 in the telephone group. The rate of missed appointments was similar in the text-message group (11.7%, 95% CI: 10.6-12.8) and in the telephone group (10.2%, 95% CI: 9.2-11.3 p = 0.07). However, only text message reminders were cost-effective. No patient reported any disturbance by any type of reminder in the satisfaction survey. Three quarters of surveyed patients recommended its regular implementation in the clinic.

**Conclusions:**

Text-message reminders are equivalent to telephone reminders in reducing the proportion of missed appointments in an academic primary care clinic and are more cost-effective. Both types of reminders are well accepted by patients.

## Background

Missed appointments are a frequent problem in outpatient clinics. They interfere with adequate medical care, misspend administrative and medical resources, and are associated with adverse patient health outcomes [1,2]. Missed appointments also appear to generate negative stereotypes of patients in primary care physicians [3]. Various interventions using different reminder methods have been tested to reduce the rate of non-attendance. Postal reminders are effective, but costly, and their effect decreases with time [4,5]. Clinical trials showed evidence that telephone reminders can reduce missed appointments [6-8]. Systematic reviews indicate that text-message reminders are more effective than no reminder among a wide age range of patients [9,10].

Only a few randomized controlled studies compared telephone and text-message reminders in medical settings and results were controversial. Two Asian studies showed that they were equally effective in primary care settings but sample sizes were small [11,12]. One study in the US showed superiority of telephone reminders over text-messaging in academic outpatient specialty clinics [13]. Little is known about the effectiveness of telephone and text-message reminders in large primary care clinics, especially in Europe. The advantages of text-message reminders are their cheaper cost and lower use of resources [12]. Still, its efficacy depends on the penetration rate of mobile phones and may therefore vary from one context to the other.

In a previous study conducted in 2008, we tested an intervention during which all patients booked into our primary care clinic were sent a reminder 48 hours prior to their appointment according to the following sequence: a phone call reminder; if the patient could not be reached by phone, a text-message reminder followed; if no mobile phone number was available for text-messaging, a postal reminder was sent. This sequential intervention significantly reduced the rate of missed appointments from 11.4% to 7.8% and was cost-effective [14]. In this study, risk factors for missing an appointment were younger age, male gender, follow-up appointment > 1 year, substance abuse and being an asylum seeker. One year after the intervention, the rate of missed appointments rose back to 14%, which called for the implementation of systematic reminders in our clinic.

As the mobile phone penetration in our population was rising (70% in 2011 versus 51% in 2008), the present study aimed to test the hypothesis that text-message reminders would be as effective as telephone reminders in a Swiss academic primary care clinic. To our knowledge, this is the first large randomized trial assessing these reminders in a primary care clinic serving a multicultural and vulnerable population. In addition, we assessed patients’ acceptance of and preference for the reminders tested.

## Methods

### Design, setting and participants

We conducted a randomized controlled non-inferiority trial in the division of primary care medicine of the Geneva University Hospitals in Switzerland. This division provides 13’000 medical consultations a year to an urban population and is a training center for 40 junior primary care doctors. It is known to care for vulnerable patient populations including undocumented migrants, asylum seekers, patients without proper insurance coverage and legal immigrants. A previous study showed that half of the patients attending the primary care clinic were immigrants and that 40% of patients did not speak French [15]. Two clinics of the division took part in the study: a general primary care clinic including migrant care and a substance abuse unit for patients with tobacco, alcohol and other substances abuse.

The rate of missed appointments varied between 12 and 14% at the general primary care clinic and between 25 and 30% at the substance abuse unit during the 6 months prior to the study. From November 2010 to April 2011, all patients registered for an appointment and for whom a cell phone number was available were invited to participate. They were randomly selected to receive a text-message or a telephone call reminder one day before the planned appointment. Patients were eligible each time they had an appointment, and were randomized each time to either reminder options. They were informed of the study by signposting in the waiting rooms and at the reception desk and orally when the appointment was made. Patients wishing to be excluded were invited to inform the receptionists. The study was approved by the ethical committee of the Geneva University Hospitals.

### Sample size

We hypothesized that text messaging reminders would not be inferior to telephone reminders in reducing the rate of missed appointments. We defined non-inferiority as less than 2% difference in the rate of missed appointments (with the missed appointment rate following telephone reminders predicted at 7.5%, on the basis of our previous study) with a power of 0.80 and p < 0.05. It required including 3151 consultations in each arm of the study. A non inferiority criterion was used in order to make the trial manageable in terms of sample size. The choice of the non inferiority margin was the result of a consensual discussion among the team and of the Head of the division. We decided that a difference of less than 2% would not bring sufficient financial or administrative benefits to justify the choice of one method over another.

### Interventions

#### Text-messaging reminders

We used “Easy SmartCare”, a software product developed by EasyMed, Services Inc, a Swiss firm specialized in the secure exchange of information and services between care providers and patients by mobile phone [16]. Patients’ phone numbers were entered into a secured web platform which automatically sent text-message reminders 24 hours before the planned appointment, including on Sundays. The text stated: “You have an appointment on… (date) at … (time) with Dr. … (name) Please answer NO if you do not intend to come”. The message was only sent in French because its length was limited to 160 characters and because it was logistically too complicated to send a message adapted to the patient’s language, since this information was not registered at the time in the electronic agenda. Each sent text-message represented a cost of 0.07 € without any charge for the patients.

#### Telephone reminders

Telephone reminders were made by a research assistant in French, Spanish, English or Italian between 10:00 am and 3:00 pm the day before the planned appointment, except for Monday appointments, when the phone call was made on Fridays. After two attempts, the research assistant would leave a message on the message box, when available, with a similar content to the text-message. Each phone call reminder cost 0.08 €.

### Randomization

A computer-generated sequence of two numbers (1 = text messaging and 2 = telephone) was produced. The research assistant (NC) randomized consecutive patients daily into two groups on a one-to-one basis, using a printed version of the electronic appointment record.

The allocation sequence was concealed from the clinical and administrative staff. Similarly, clinical and administrative staffs were blinded after assignment to the intervention, since reminders were managed by a research assistant and the team of EasyMedmobile.

### Satisfaction survey

At the end of the study, we conducted a telephone survey to evaluate the acceptability and usefulness of the interventions among a random sample of 900 patients. The telephone survey included the following questions: 1) what type of reminder did you receive for your appointment(s)? ; 2) were you disturbed by the reminder? (distinct answers according to the type of reminder(s) received); 3) did you consider the reminder to be useful? (answers collected only for the type of reminder(s) received by the patient); 4) would you recommend the systematic use of a reminder? (answers collected for all types of reminders, whatever the reminder received). The research assistant spoke fluent French, Spanish and English and adapted the language to patients’ needs.

### Outcome measures

The primary outcome was the rate of unexplained missed appointments. Appointments that were cancelled or re-scheduled before the planned appointments were not considered as missed. The rate of cancelled or rescheduled appointments the day before and the day of the appointment as well as the number of reallocations were collected as secondary outcomes. The variables collected in the satisfaction survey are detailed in the previous section.

### Statistical analysis

Stata software was used for the analysis. Analyses were performed by a researcher (DHH) who was neither involved in the study implementation or in the data collection, nor in the administrative routine work of the clinic or in patient care. The statistical analysis was conducted according to the “intention to treat” principle and included all appointments once randomization had occurred, excluding appointments that were cancelled before or independently from the intervention. We compared patient and health care providers’ baseline characteristics between groups by means of Chi square tests for categorical variables and Student’s-*t*-test for continuous variables. We compared the rate of missed appointments between both groups and calculated odds ratios and confidence interval. P values of 0.05 or less were considered statistically significant.

## Results

**Figure 1:**
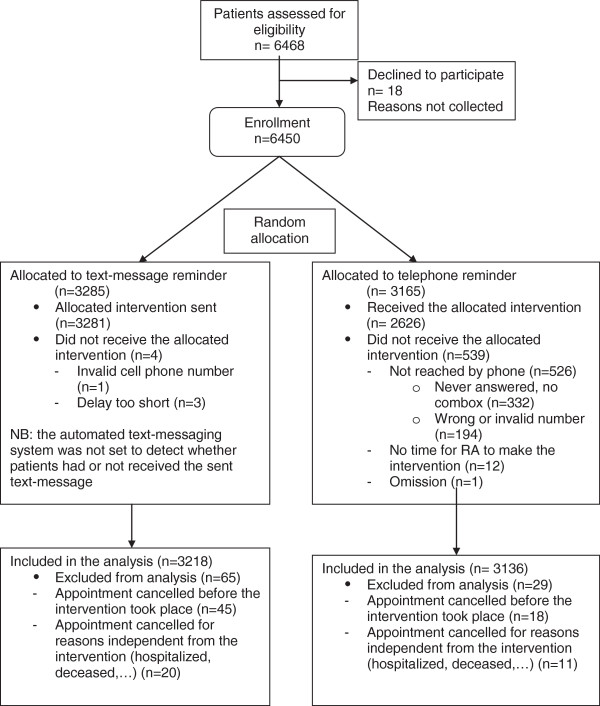
Participant flow chart.

Out of 6468 planned appointments, 6450 were eligible for randomization: 3285 in the text-message reminder arm and 3165 in the telephone reminder arm. The randomization process and the patients’ flow are displayed in Figure 1.

Among the 6354 analyzed appointments, 78% came from the general primary care clinic in the text messaging group and 79.6% in the telephone group. Both arms were comparable in term of patients’ age and gender and of health professionals’ profile (Table 1).

**Table 1:** Sample characteristics (N = 6433, missing data n = 17)

	**Text-message reminder group**	**Telephone reminder group**	**p**
Patient mean age (yrs) (SD)	44.2 (12.4)	44.5 (12.7)	0.28
Patient gender, female n (%)	1732 (53.1)	1736 (54.8)	0.17
Health professional status n (%)			
- Junior doctor	2422 (74.2)	2313 (73.0)	0.06
- Senior doctor	769 (23.6)	805 (25.4)	
- Psychologists or psychiatrist	37 (1.1)	20 (0.6)	
- Nurse	36 (1.1)	30 (0.9)	

During the interventions, the overall rate of missed appointments in the division was 11%, with higher rates in the substance abuse unit (17%) than in the general primary care clinic (9.3%) (Table 2). The rate of missed appointments was similar in the text-message group (11.7%) and in the telephone group (10.2%). Telephone reminders were slightly and significantly more effective to reduce missed appointments than text messaging in the general primary care clinic, but did not reach the 2% non-inferiority definition. The effect of both types of reminders was similar in the substance abuse unit. No fatigue effect was observed over the entire duration of the study (Table 3).

**Table 2:** Rate of missed appointments (n = 6354)

	**Total**	**Text-message reminder**	**Telephone reminder**	**Relative effect size**	**P value**
	**n (%)**	**n (%)**	**n (%)**	**OR (IC)**	
Division of primary care	697 (11.0)	376 (11.7)	321 (10.2)	0.9 (0.7-1.0)	0.07
- General primary care clinic	468 (9.3)	256 (10.2)	212 (8.5)	0.8 (0.7-1.0)	0.04
- Substance abuse clinic	229 (17.0)	120 (17.1)	109 (17.0)	1.0 (0.7-1.3)	0.98

**Table 3:** Monthly rate of missed appointments in the text-message and telephone reminder arms during the study period

**Month**	**n**	**Text-message reminder**	**Telephone reminder**	**P value**
		**%**	**%**	
Nov 2010	400	11.4	7.7	0.22
Dec 2010	1112	13.1	9.6	0.07
Jan 2011	850	14.0	11.4	0.25
Feb 2011	1520	11.7	10.5	0.44
March 2011	1518	9.8	10.4	0.70
April 2011	948	10.9	9.8	0.60

The rate of appointments cancelled or rescheduled by patients between reception of the reminder and the consultation (the day before and the day of the appointment) was 6.1% (4.9% cancelled appointments and 1.2% rescheduled appointments) for the overall division, with no difference between text-messaging and telephone groups. In the general primary care clinic, rates of cancelled appointments were similar: 4.8% in the telephone group versus 3.9% in the text-messaging (p = 0.267). In the substance abuse unit, rates of cancelled appointments were statistically higher in the text-messaging group than in the telephone group (8.8% vs. 5.9%, p = 0.044). The rates of rescheduled consultations were the same in both settings and in both the telephone and text messaging groups (1.1-1.3%). No free slots were reallocated.

Table 4 shows that although both reminders were effective in reducing the rate of missed appointments, only the text-message reminder system was cost-effective, mostly because additional administrative resources were needed for the telephone reminders. Costs were calculated on the basis of current telecommunication rates in our setting and on a 30% daily administrative activity five days a week to make the phone calls.

**Table 4:** Cost-comparisons between both types of reminders based on current telecommunication costs in Switzerland (in Euros)

	**Items**	**Text-message reminder group**	**Telephone reminder group**
**COST**			
Telecommunication cost			
(0.07 €/text-message)	3281 appointments	230.-	
(0.08 €/phone call)	2626 appointments*		210.-
Administrative work to make manual phone calls during 6 months (30%) (€)			8’700.-
Total costs (€)		230.-	8910.-
**BENEFITS**			
Nr of additional appointments attended compared to the 14% rate of missed appointments before the intervention (Fees =80(€) per consultation)	84 for text message	6’720.-	
	122 for telephone		9’760.-
Net benefits (€)		6490.-	850.-

* Patients reached by the telephone reminder.

Out of 900 patients randomly selected for the survey, 41% were reached after 2 attempts. Both types of reminders were very well accepted by responders. Three quarters of surveyed patients recommended its systematic use. However, a higher percentage of patients preferred text-message reminders to phone call reminders, especially among patients from the substance abuse unit (Table 5).

**Table 5:** Satisfaction survey among a sample of patients having received the intervention reminders

	**General primary care clinic (n = 288)**	**Substance abuse clinic (n = 85)**
	**n (%)**	**n (%)**
Type of reminder		
- Text messaging	89 (30.9)	35 (41.2)
- Telephone	104 (36.1)	18 (21.2)
- Text messaging and telephone	95 (33.0)	32 (37.6)
I was disturbed by the reminder		
- Text messaging	1 (0.5)	2 (3.0)
- Telephone	0	5 (10.0)
The reminder is useful		
- Text messaging	181 (98.4)	60 (88.2)
- Telephone	196 (98.5)	42 (85.7)
I recommend the use of a systematic reminder		
- Text messaging	211 (77.6)	74 (88.1)
- Telephone	192 (70.3)	40 (52.6)

## Discussion

To our knowledge, this study is the first large study to report the effectiveness of text-messaging versus telephone reminders in a primary care setting in Europe. Our findings confirm the non-inferiority of text messaging versus telephone reminders to reduce the rate of missed appointments in an academic primary care clinic and the cost-effectiveness of text-message reminders compared to manual phone reminders. Both types of reminders were well accepted by all patients, but text-messages were preferred, particularly among patients consulting for a tobacco, alcohol or other substance abuse.

Rates of attendance were slightly higher following telephone reminders than text-message reminders among general primary care patients, indicating that direct personal contact with the patient may be more effective than a machine-generated message. This is consistent with another study which showed that telephone reminders by a staff member were also slightly more effective than automated phone calls to reduce non-attendance in an academic outpatient practice [13]. However, these findings were not confirmed in another study assessing the effectiveness of automated versus staff phone reminders for colonoscopies [17]. Patients’ perceptions may differ according to the type of health care setting and care provision. The difference in the rate of attendance between both groups was smaller than 2%, our pre-defined non-inferiority margin. The benefits of a higher attendance with telephone reminders can thus be considered minimal.

The fact that a higher percentage of patients in the substance abuse unit cancelled appointments following a text-message rather than a telephone reminder suggests that this particular group of patients may be more receptive to text-messaging than telephone reminders. It is well known that non-attendance rate among patients with mental disorders is high (up to 40%) [18,19], predominantly among patients with alcohol and drug abuse (18-36%) [20]. This is probably linked to the fact that patients suffering from substance abuse or mental disorders in general may have increased socio-economic difficulties and impairment complicating their regular access to care [18,21]. Also, motivation for substance abuse care can fluctuate between the time of scheduling and the time of appointment. We found no comparative data on the effectiveness of telephone or text-messaging reminders in this particular population of primary care attenders. Contrary to our expectations, no slots were reallocated after appointments had been cancelled in response to the reminder. This might be due to the short delay (24 h) between the reminder and the consultation itself. In a previous study, 48 hours delay allowed reallocation of 28% of the spaces made vacant after cancellation [14]. Increasing the reminder delay and improving the ability to identify vacant spaces in our electronic appointment system may also help reallocate these free appointments more efficiently in the future. However, although both reminders were equally effective in reducing the rate of missed appointments, only the text-message system was cost-effective, because of the absence of additional administrative work. Further research should explore the non-inferiority of text-messaging reminder compared to automated phone call reminders.

As in previous studies on telephone and text-message reminders, both types of reminders were well accepted by a large majority of patients [13,17,22]. Only very few patients declared that they had been disturbed by the intervention, although those who opted out might have done so because they judged the intervention would be disturbing in the first place. However, their number was minimal as shown in the flow chart. We are unable to explain why substance abuse patients showed a preference for a text-message over a phone reminder. However, such preference should be taken into consideration since text-messaging was associated with a higher rate of cancelled appointments.

One negative effect of reminders, whether phone or text-message, is that they shift the responsibility of attendance away from the patient to the organization [23]. Alternative effective interventions include incentives, such as free treatment for patients with regular attendance [24], or compulsory involvement in an educational program in order to continue receiving care after several missed appointments [25]. Given the high number of missed appointments and the difficulty to define a specific profile of non-attenders, large scale implementation of such interventions does not appear to be realistic, at least in our context.

Our study had some limitations. It was a single-center study conducted in an academic primary care clinic providing care to patients with low socio-economical status with a rapid turnover of physicians. Results may not be generalizable to other settings although they are consistent with other studies. Since the main outcome variable was the appointment and not the patient, we did not collect information about the number of reminders sent to each patient during the main study. The automated software was not set to provide information on whether patients had received the text message or not. Therefore, we did not collect information about the estimated rate of patients reached by the text-message. The fact that text messages were sent in French only whereas phone reminders occurred in four languages may have enhanced the effectiveness of telephone reminders over text messages. The satisfaction survey also had several limitations: patients’ acceptance of reminders was assessed in a limited sample of them, and only among those who could be reached by phone during the survey; we only asked patients which type of reminder they had received, but did not ask them about the number of reminders received; we did not record the number of patients who reported not having received a text message or a phone call reminder. Costs in our cost-effectiveness analysis represent charges. A more detailed cost-effectiveness analysis including purchase and maintenance costs was not possible, since we could not assess the costs corresponding to the purchase and to the maintenance of the central telephonic switchboard of our division and of our hospital, and of an automated text messaging system. Nevertheless, maintenance costs of both systems are probably equivalent. Finally, we did not collect data about misreading/misinterpretation of written or oral information, issues of privacy and disclosure [9].

## Conclusions

In conclusion, text-messaging reminders appear to be as effective as phone reminders to decrease the rate of missed appointments and are more cost-effective. Such a reminder system seems ideal, as many electronic appointment systems can generate automatic text-message reminders at very low cost. The next step will be to assess whether adding information in text-message reminders may improve patients’ compliance with tasks to be performed before the next consultation (such as blood tests, medication intake or blood pressure control).

## Competing interests

The authors declares that they have no competing interests.

## Authors’ contributions

NJP conceived and designed the study, coordinated the data collection and wrote the first draft of the manuscript. MDD participated to the study coordination and the manuscript writing. NCR collected the data and critically revised the manuscript. JPH, BB, FN participated to the design and coordination of the study and critically revised the manuscript. DH participated to the design study, carried out the analysis and contributed to the manuscript writing. JMG helped to the design and funding of the study and critically revised manuscript. All authors read and approved the final manuscript.

## Pre-publication history

The pre-publication history for this paper can be accessed here:

http://www.biomedcentral.com/1472-6963/13/125/prepub
